# Caught in the Crossfire: How Contradictory Information and Norms on Social Media Influence Young Women’s Intentions to Receive HPV Vaccination in the United States and China

**DOI:** 10.3389/fpsyg.2020.548365

**Published:** 2020-12-03

**Authors:** Shuya Pan, Di Zhang, Jingwen Zhang

**Affiliations:** ^1^Renmin University of China, Beijing, China; ^2^University of California, Davis, Davis, CA, United States

**Keywords:** contradictory information exposure, social norms, social media, vaccination intention, HPV vaccine

## Abstract

This study uses online survey data from the United States and China to examine how contradictory information and social norms regarding HPV vaccines obtained through social media are related to young women’s attitudes and intentions surrounding HPV vaccination. The results show that exposure to contradictory information on social media had a greater negative association with intentions to receive HPV vaccination among the United States participants than among the Chinese participants, while social norms supporting HPV vaccines had a stronger positive association with intentions to receive HPV vaccination among the Chinese participants than among the United States participants. These findings extend the literature on social media communication regarding HPV vaccination and contribute to our knowledge of cultural contexts that influence intentions to receive HPV vaccination.

## Introduction

Human papilloma virus (HPV) is the most common sexually transmitted infection (STI) and is the primary cause of cervical cancer and other HPV-related cancers in women and men (Centers for Disease Control and Prevention, n.d.). Recent developments in vaccines targeting high-risk types of HPV have helped decrease the risk of developing HPV-related diseases and cancer precursors (Joura et al., 2015). Since 2006, several countries have implemented publicly funded HPV immunization programs (Bruni et al., 2016). More than a decade later, however, the uptake of HPV vaccination among adults aged 18–26 years in the United States (U.S.) remains low (Brooks, 2020, January 7). There are multiple reasons for such underuse, including stigma associated with a vaccination against STIs, perceived risks, and emerging antivaccine sentiment (Katz et al., 2011; Shepherd and Gerend, 2014; Dubé et al., 2015).

As social media provide tremendous opportunities for people to exchange information and personal opinions related to health issues, including HPV vaccines (Massey et al., 2016), recent studies show that exposure to social media messages can shape young female social media users’ understanding of and attitudes toward HPV vaccines and their vaccination decisions (Dunn et al., 2017). Specifically, some studies suggest that social media users can be exposed to a large volume of contradictory messages related to various health topics, including HPV vaccination (Shelby and Ernst, 2013; Zimet et al., 2013). In this study, contradictory information is defined as statements that are logically inconsistent (Carpenter et al., 2016). Additionally, exposure to social media messages helps create normative perceptions of a particular behavior (Zeng et al., 2009; Chu and Kim, 2011). Thus, the current study explores how contradictory messages and perceived norms on social media are associated with intentions to receive HPV vaccination among young women aged 18 to 25 years, the most avid social media users.

Additionally, this study applies a comparative approach to examine how the U.S. and China differ in the association between HPV vaccination-related contradictory messages/norms on social media and intentions to receive HPV vaccination. The HPV vaccine was not approved in mainland China until 2016, although the incidence and mortality rates of cervical cancer continue to increase (He and He, 2018). Interestingly, in contrast to the vaccine hesitancy found in the U.S., studies have documented a high level of acceptance and willingness to receive HPV vaccines among Chinese women (J. Li et al., 2009; W. Li et al., 2018). Underlying these contrasts are fundamental differences in culture, health care systems and health policies (Chen, 2001). The findings of this study extend the literature on social media communication regarding the HPV vaccine and significantly contribute to our knowledge of sociocultural differences in mechanisms associated with the intention to receive HPV vaccination.

More importantly, this study contributes to research on health communication from the perspective of cultural psychology. Traditionally, cultural psychology focuses on the effects that individual-level cultural beliefs and values have on people; however, cultural psychologists also hold that supra-individual contexts play an important role in shaping people’s behaviors (Chiu et al., 2010). Regarding culture, China is more collectivistic and has a higher tolerance for uncertainty than the U.S. (Hofstede, 2001). The magnitudes of normative influences and reaction to contradictions are related to degrees of individualism versus collectivism and to tolerance for uncertainty, respectively (Triandis, 1995; Spencer-Rodgers et al., 2010). Although this study does not directly measure cultural norms at the individual level, the differential strength of the relationships between contradictory messages/norms regarding the HPV vaccination and vaccination behaviors on social media, if any, may reflect the effects of cross-cultural differences and guide future studies of the precise mechanisms through which cultural beliefs, at either the individual or supra-individual level, influence health behaviors.

## Literature Review

### Exposure to Contradictory Health Information on Social Media

Contradictory information has been defined as statements that are logically inconsistent (Carpenter et al., 2016). Such inconsistencies can be found in true and false messages and in scientifically approved positive and negative findings on an issue.

For example, social media sites are rife with myths and anecdotes about the HPV vaccine, including accounts of severe side effects and HPV infection (Briones et al., 2012). Such misinformation, which is defined as a claim of fact that is “currently false due to a lack of scientific evidence” (Chou et al., 2018), can contradict scientific findings and lead to harmful or even dangerous health behaviors and decisions (Lewandowsky et al., 2012; Zimet et al., 2013; Tan et al., 2017).

Another line of research shows that contradictions can be present in messages containing negative and positive aspects of scientific findings. HPV vaccines are effective in preventing cervical cancer but also have some side effects, such as fever and arm pain. Research has shown that exposure to such two-sided health information can increase the ambivalence of a message and spur reluctance to act on health recommendations (Chang, 2013).

Uncertainty management theory (UMT, Brashers, 2001) holds that uncertainty emerges from ambiguous and complex information. Contradictory messages do not provide clear information or guidance that can allow individuals to adequately structure or categorize an object (HPV vaccines and vaccination, in the context of this study); in other words, since information cues are insufficient or insufficiently cogent, individuals cannot reliably assign meaning to an object or accurately predict its outcomes (Mishel, 1988, 1990), which leads to confusion and negative beliefs about the object in the context of health communication (Nagler et al., 2019).

In addition, previous research has measured contradictory information in different ways. Some works have used experimental methods to assess forced exposure to controlled contradictory information (Dixon and Clarke, 2012; Nan and Daily, 2015). For example, Nan and Daily (2015) designed different conditions of HPV vaccine-related messages posted on blogs and found that a mix of positive and negative information about HPV vaccines provided in blog posts can lead to polarized vaccine-related beliefs. Another set of research holds that one’s subjective evaluation of contradictions in information matters more than the actual level of contradiction controlled in messages (Nagler, 2014; Lee et al., 2018). For instance, Lee et al. (2018) found that subjective perceptions of exposure to contradictory nutrition information decrease people’s fruit and vegetable consumption. As people from different countries and cultures might differ their interpretation of the actual levels of contradiction in messages, we decided to use a subjective means to measure perceptions of contradiction in HPV vaccine-related messages. Thus, the following hypotheses are proposed:

H1a: Greater exposure to contradictory HPV vaccine messages on social media is associated with a more negative attitude toward HPV vaccination.
H1b: Greater exposure to contradictory HPV vaccine messages on social media is associated with lower intentions to receive HPV vaccination.

## Perceived Norms of HPV Vaccination on Social Media

In social psychology, social groups, family members, and peers are generally believed to be significant influencers of an individual’s attitudes and intentions (Prislin and Wood, 2005). Such influence may occur when people make inferences about their peers’ thoughts and behaviors based on their social media activities. These perceptions of others fall into the theoretical territory of normative social influences (Nolan et al., 2008; Tankard and Paluck, 2016; Bicchieri, 2017). In both social and cultural psychology, norms are an important concept that has gained considerable attention. The theory of planned behavior (TPB), a well-established behavior theory, emphasizes the effects of subjective norms on attitudes, which lead to behavioral intentions and behaviors (Ajzen, 1991). The original TPB defined subjective norms as “perceived social pressure to perform or not to perform the behavior” (Ajzen, 1991, p. 188) and was believed to capture the essence of injunctive norms, conceptualized as perceptions of the extent to which significant others approve or disapprove of a given behavior (White et al., 2009). Other studies reveal that in addition to subjective or injunctive norms, descriptive norms, which assess perceptions of others’ attitudes or behaviors, can explain variations in behaviors (Rivis and Sheeran, 2003). In cultural psychology, descriptive norms also refer to intersubjective norms or consensus (Gelfand and Harrington, 2015); in addition to acting on their internalized beliefs and values, people act on the beliefs and values they perceive as being shared in their culture (Chiu et al., 2010).

Much existing research operationalizes normative perceptions in reference to individuals’ offline social contacts, such as their parents and friends (Rivis and Sheeran, 2003; Rimal and Real, 2005; Rimal, 2008; McEachan et al., 2011; Padon et al., 2016). Instead, this study focuses on the effects of injunctive and descriptive norms inferred from an individual’s exposure to information through social media. Beyond diffusing health messages, social media also give rise to forces that form and change health and behavior norms. Through social media, people most often connect with others with similar backgrounds and characteristics, and indeed, some online connections overlap with people’s real-life connections (Reich et al., 2012). Individual social media users’ expressions and presentations of their own behaviors constantly send signals to their followers and to other users who read their posts, which may form and change those readers’ social perceptions (Yoo et al., 2016). Research has demonstrated that social norms, as perceived on social media, can impact individuals’ health-related attitudes and behavioral intentions in terms of drinking behaviors (Fournier et al., 2013; Geusens and Beullens, 2016, 2017). It has also been found that exposure to sexually suggestive Facebook photos leads college students to estimate that a higher percentage of their peers are engaging in risky sexual behaviors and to report a higher intention to engage in the same behaviors themselves (Young and Jordan, 2013).

This study measures both injunctive and descriptive norms. Injunctive norms on social media refer to a person’s perceptions of approval of HPV vaccination among those with whom he or she interacts on social media; descriptive norms on social media refer to a person’s perceptions of the prevalence of HPV vaccine uptake among those with whom he or she interacts on social media. Thus, we propose the following hypotheses:

H2a: Descriptive norms on social media are positively associated with attitudes toward HPV vaccination.

H2b: Descriptive norms on social media are positively associated with behavioral intentions to receive HPV vaccination.

H3a: Injunctive norms on social media are positively associated with attitudes toward HPV vaccination.

H3b: Injunctive norms on social media are positively associated with behavioral intentions to receive HPV vaccination.

## The Mediating Role of Attitude

Traditionally, attitudes have been viewed as a major variable that explains the intention to perform an action within the “attitude-intention-behavior model” (Ajzen, 1991; Ajzen and Fishbein, 1977) of social psychology. Jolley and Douglas (2014), for instance, found a significant negative relationship between antivaccine attitudes and vaccination intentions. Thus, the following hypothesis is proposed:

H4: Attitudes toward HPV vaccination are positively associated with behavioral intentions to receive HPV vaccination.

**FIGURE 1:**
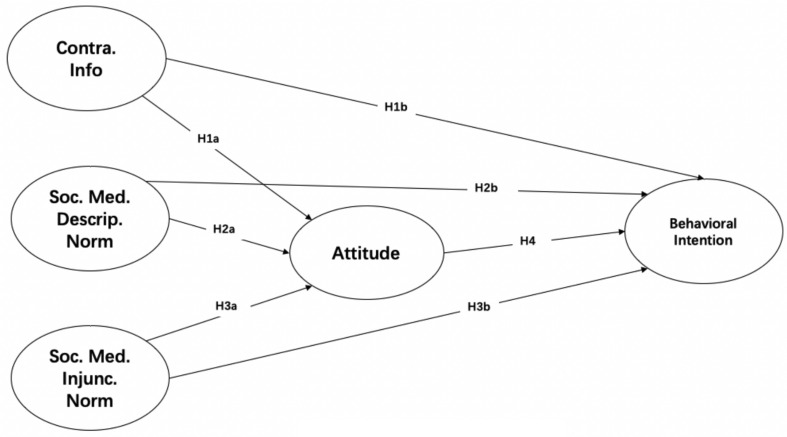
Conceptual model.

In summary, this study proposes the research model illustrated in Figure 1. Contradictory messages and norms (descriptive and injunctive) on social media are associated with attitudes toward HPV vaccination. Attitude acts as a mediator between contradictory messages (and norms) on social media and behavioral intentions.

## Cross-Country and Cultural Differences

There is an increasing call for comparative cross-country/cross-cultural health communication research (e.g., Kreps et al., 2017) as health communication is inherently culture bound (Dutta, 2007). Major health communication theories and models have emerged from research conducted primarily in Western countries. However, Chinese culture has its own unique health beliefs, behavioral patterns and practices, which differ from those of Western countries (Jing et al., 2013; Yuan, 2017). For instance, many Chinese people believe in Chinese traditional medicine based on a holistic worldview that values everyday health management using certain diet therapies, herbal remedies and exercises (Rawl, 1992; Yuan, 2017). Additionally, Chinese people are less likely to share their experiences of socially stigmatized health problems with others and are more likely to be influenced by family members and friends when making health decisions (Chen, 2001).

Cultural dimension theory (Hofstede et al., 2010) suggests that countries/cultures differ from one another in power distance, individualism-collectivism, masculinity-femininity, uncertainty avoidance and long-term orientation. Rather than pinpointing how these cultural beliefs influence people’s health behaviors at the individual level, this study explores the possible link between supra-individual level contexts (Chiu et al., 2010) and the formation of health attitudes and behavioral intentions. Although this study does not measure cultural beliefs at the individual level, the literature suggests that differences in individualism-collectivism and uncertainty avoidance between the U.S. and China might lead people to react differently when facing contradictory messages and normative influences on social media (Triandis, 1995; Spencer-Rodgers et al., 2010).

Uncertainty avoidance measures the extent to which the members of a culture feel threatened by ambiguous or unknown situations (Hofstede et al., 2010). The most recently published Hofstede Insights index shows that Americans have less tolerance for uncertainty (46) than Chinese people do (30). Studies on tolerance of contradiction further suggest that individuals from East Asian cultures are more inclined to tolerate contradiction (Peng and Nisbett, 1999; Spencer-Rodgers et al., 2009). A related concept of contradiction holds that beings in the universe consist of opposing elements (Spencer-Rodgers et al., 2010) and that the universe is in a state of constant change, such that what is false today may become fact tomorrow and vice versa (Spencer-Rodgers et al., 2010), and what is considered right and wrong is specific to certain contexts and situations (Spencer-Rodgers et al., 2010). Thus, contradiction ought to be tolerated, and exposure to contradictory HPV vaccine messages on social media might have smaller impacts on the Chinese population.

Furthermore, in individualistic cultures such as the U.S., social behaviors are largely determined by personal goals and values; collectivistic cultures such as China are more influenced by the goals and values shared among collectives (Mills and Clark, 1982; Triandis, 1988). It has been found that norms are less related to behavioral intentions in more individualistic cultures (Fischer and Karl, 2020, February 11). Thus, in collectivistic cultures, social norms on social media, like those in real life, might exert more influence on people. It is reasonable to expect Chinese and American people to be affected differently by norms on social media. From this discussion, the following research question is posed:

RQ: How do the U.S. and China differ in the associations between HPV vaccination-related contradictory messages/norms on social media and behavioral intentions via attitudes toward HPV vaccination?

## Methods

### Sample

We conducted cross-sectional online surveys of both U.S. and Chinese samples. The institutional review board of the university with which the researchers are affiliated approved the study’s protocols, and the participants were provided a consent form prior to completing the survey.

For both the U.S. and Chinese samples, eligible participants had to be female, be 18 to 25 years of age and not have received the HPV vaccine. Additionally, this study, which focuses on the contradictory messages and normative influences of social media, only recruited subjects who are active social media users and who have encountered information on the HPV vaccine on social media. The researchers asked, “How often do you encounter HPV vaccine-related information on social media?” and excluded subjects who selected “Never.”

For the U.S. sample, an online survey link generated by the Qualtrics platform was distributed to qualified participants via Amazon Mechanical Turk (MTurk) in August 2018. Among 264 eligible U.S. participants, 133 who reported having been exposed to HPV vaccine-related information on social media were included in the study. The results of the power analysis of the overall test of model fit based on the RMSEA suggest a minimum sample size of 118 (RMSEA = 0.05, *df* = 181, power = 0.80), proving that our sample was more than adequate. As Table 1 shows, for the U.S. sample, the average age of the participants was approximately 22 years (*M* = 22.21, *SD* = 2.11). The majority of the U.S. participants had not obtained a bachelor’s degree (*N* = 94; 70.68%), and more than half (*N* = 87; 65.41%) were not single. Most of the participants were employed (*N* = 55; 41.35%) and Caucasian (*N* = 92; 69.17%). Most of the U.S. participants reported having had sexual intercourse (*N* = 99; 74.44%).

**TABLE 1:** Descriptive statistics of respondents.

	U.S. sample (*N* = 133)				Chinese sample (*N* = 254)			Mean diff.		Pooled sample (*N* = 387)		
Variables	M	SD	*n*	%	M	SD	*n*	%		M	SD	*n*
Age	22.21	2.11			21.81	1.94				21.95	2.00	
**Education**												
Less than a bachelor’s degree			94	70.68			35	13.78				129
Bachelor’s degree and above			39	29.32			219	86.22				258
**Relationship status**												
Single			46	34.59			139	54.72				185
In relationship			87	65.41			115	45.28				202
**Occupation**												
Employed			55	41.35			110	43.31				165
Working part-time			34	25.56			60	23.62				94
Unemployed			44	33.08			84	33.07				128
**Ethnicity**												
White			92	69.17								
Non-white			41	30.83								
**Sexually active**												
Yes			99	74.44			90	35.43				189
No			34	25.56			164	64.57				198
Exposure*	3.32	2.41			3.92	1.91			−0.60**	3.71	2.11	
SMDN*	11.98	3.46			14.17	3.66			−2.19**	13.42	3.73	
SMIN*	12.86	3.68			15.03	3.71			−2.17**	14.28	3.84	
AT*	19.91	6.96			22.20	4.24			−2.29**	21.41	5.43	
BI*	13.20	5.69			16.41	3.67			−3.21**	15.30	4.71	

*Exposure, exposure to contradictory messages regarding the HPV vaccine on social media; SMDN, descriptive norms on social media; SMIN, injunctive norms on social media; AT, attitudes toward the HPV vaccination; BI, behavioral intentions to receive the HPV vaccine; M, mean; SD, standard deviation; N, number;%, percent. **Mean difference is calculated by the mean differences in exposure, SMDN, SMIN, AT and BI between the U.S. respondents and the Chinese respondents. All the pairwise comparisons of exposure to contradictory messages, SMDN, SMIN, attitudes and behavioral intentions between the Chinese and U.S. respondents are statistically significant at *p* < 0.05. Results of *t*-tests are also reported in the results section of the text.

The Chinese sample was recruited through the Chinese online panel WJX.CN in August 2018. In total, 285 eligible cases were recruited, and 31 cases were excluded because they had never been exposed to HPV vaccine-related information on social media. As Table 1 shows, for the Chinese sample, the average age was also approximately 22 years (*M* = 21.81, *SD* = 1.94). The majority of the participants reported having a bachelor’s degree or above (*N* = 219; 86.22%). Slightly more than half of the Chinese participants were single (*N* = 139; 54.72%). Most of the participants were employed (*N* = 110, 43.31%) and reported not having had sexual intercourse (*N* = 164; 64.57%).

### Measures

#### Exposure to Contradictory Messages

Exposure to HPV vaccine-related contradictory messages on social media was measured with one item asking the participants to rate the amount of contradictory HPV vaccine-related information they had viewed on social media on a scale from 0 (none) to 10 (very much).

#### Social Norms on Social Media

Measures of social norms on social media were adapted from Park and Smith (2007). Descriptive and injunctive norms were measured with three items each, rated on a scale from strongly disagree (1) to strongly agree (7). The sample item for descriptive norms was “Most girls/women I communicate with on social media would consider receiving the HPV vaccine.” The sample item for injunctive norms was “Most girls/women I communicate with on social media would approve of me receiving the HPV vaccine.” For the Chinese sample, the Cronbach’s alpha for descriptive norms was0.86, and that for injunctive norms was 0.89; for the U.S. sample, these values were 0.91 and0.94, respectively.

#### Attitudes Toward HPV Vaccination

Using a seven-point semantic-differential scales adapted from Lipkus et al. (2001), the subjects were asked to rate their attitudes toward “receiving the HPV vaccine” on the following four levels: bad/good, unwise/wise, unfavorable/favorable, and harmful/beneficial (China: Cronbach’s α = 0.89; U.S.: Cronbach’s α = 0.97).

#### Behavioral Intention to Receive the HPV Vaccine

The intention to receive the HPV vaccination was measured using the following three items adapted from Gerend and Shepherd (2007): “How likely are you to (a) consider receiving; (b) try to obtain; or (c) actually receive the vaccine once it is available?” Responses were recorded on a seven-point scale ranging from extremely unlikely (1) to extremely likely (7) (China: Cronbach’s α = 0.87; U.S.: Cronbach’s α = 0.97).

### Data Analysis

Structural equation modeling was employed to test the hypotheses and examine the research question via the Lavaan package in R. The research question was answered using multigroup structural equation modeling.

## Results

In this section, descriptive statistics are presented first. To examine the seven hypotheses, measurement and path models for the pooled samples were built. Then, the researchers evaluated the metric equivalence of the U.S. and Chinese samples before constructing a multigroup structural model. Finally, a multigroup structural model comparing the Chinese and U.S. samples was assessed.

### Descriptive Statistics

As Table 1 shows, the Chinese respondents reported higher scores for both descriptive and injunctive norms on social media than their U.S. counterparts did (*t*_SMDN_ = 5.78, *p* < 0.05; *t*_SMIN_ = 5.50, *p* < 0.05), and they encountered higher exposure to contradictory information on social media than the U.S. respondents did (*t*_exposure_ = 2.46, *p* < 0.05). Furthermore, the Chinese respondents had more positive attitudes toward HPV vaccination than the U.S. respondents did (*t*_attitude_ = 3.47, *p* < 0.05). Finally, the Chinese respondents reported greater intentions to receive HPV vaccination than their U.S. counterparts did (*t*_intention_ = 5.91, *p* < 0.05).

Tables 2 and 3 present the correlation coefficients among variables potentially associated with the intention to receive the HPV vaccination, including attitudes toward HPV vaccination, norms perceived through social media, and contradictory HPV vaccination information exposure through social media. These results suggest that China and the U.S. differed in the bivariate relationship between vaccination intentions and contradictory information exposure/norms perceived through social media/attitudes toward vaccination. For instance, the association between intentions and attitudes/contradictory information exposure was stronger for the U.S. sample than for the Chinese sample. The relationship between intentions and descriptive norms was slightly stronger for the Chinese sample than for the U.S. sample, while the reverse was found for the association between intentions and injunctive norms.

**TABLE 2:** Coefficients for pairwise correlation among predictor and outcome variables in the Chinese sample.

	IN2	IN3	AT1	AT2	AT3	AT4	E	SMDN1	SMDN2	SMDN3	SMIN1	SMIN2	SMIN3
IN1	0.78	0.66	0.39	0.35	0.30	0.29	−0.19	0.39	0.38	0.29	0.38	0.34	0.32
IN2		0.65	0.38	0.35	0.30	0.30	−0.17	0.38	0.40	0.28	0.40	0.37	0.34
IN3			0.41	0.39	0.30	0.30	−0.19	0.42	0.40	0.42	0.35	0.34	0.41
AT1				0.73	0.72	0.62	−0.24	0.33	0.31	0.32	0.39	0.33	0.38
AT2					0.66	0.65	−0.24	0.31	0.32	0.27	0.34	0.31	0.35
AT3						0.70	−0.21	0.30	0.24	0.22	0.30	0.27	0.26
AT4							−0.14	0.29	0.21	0.17	0.27	0.23	0.22
E								−0.18	−0.22	−0.18	−0.20	−0.19	−0.18
SMDN1									0.74	0.61	0.53	0.57	0.54
SMDN2										0.65	0.56	0.59	0.58
SMDN3											0.52	0.56	0.62
SMIN1												0.73	0.69
SMIN2													0.76

*All the coefficients are statistically significant at the level of *p* < 0.05. E, exposure to contradictory messages regarding the HPV vaccine on social media; SMDN, descriptive norms on social media; SMIN, injunctive norms on social media; AT, attitudes toward the HPV vaccination; BI, behavioral intentions to receive the HPV vaccine.*

**TABLE 3:** Coefficients for pairwise correlation among predictor and outcome variables in the U.S. sample.

	IN2	IN3	AT1	AT2	AT3	AT4	E	SMDN1	SMDN2	SMDN3	SMIN1	SMIN2	SMIN3
IN1	0.94	0.92	0.69	0.68	0.74	0.69	−0.37	0.37	0.37	0.38	0.35	0.46	0.43
IN2		0.92	0.68	0.69	0.70	0.69	−0.37	0.35	0.35	0.35	0.36	0.43	0.41
IN3			0.68	0.67	0.72	0.68	−0.38	0.33	0.35	0.38	0.34	0.43	0.43
AT1				0.91	0.89	0.89	−0.21	0.38	0.37	0.39	0.42	0.47	0.43
AT2					0.89	0.92	−0.27	0.49	0.44	0.41	0.41	0.46	0.46
AT3						0.89	−0.24	0.47	0.46	0.46	0.41	0.50	0.49
AT4							−0.28	0.47	0.41	0.40	0.36	0.42	0.43
E								−**0.16**	−0.19	−0.18	−0.19	−**0.16**	−**0.17**
SMDN1									0.76	0.70	0.55	0.67	0.64
SMDN2										0.84	0.66	0.66	0.72
SMDN3											0.71	0.75	0.77
SMIN1												0.84	0.85
SMIN2													0.84

*All the coefficients are statistically significant at the level of *p* < 0.05 except the coefficients in bold, which are only statistically significant at the level of *p* < 0.10. E, exposure to contradictory messages regarding the HPV vaccine on social media. SMDN, descriptive norms on social media; SMIN, injunctive norms on social media; AT, attitudes toward HPV vaccination; BI, behavioral intentions to receive the HPV vaccine.*

### Hypothesis Testing for Main Effects

**FIGURE 2:**
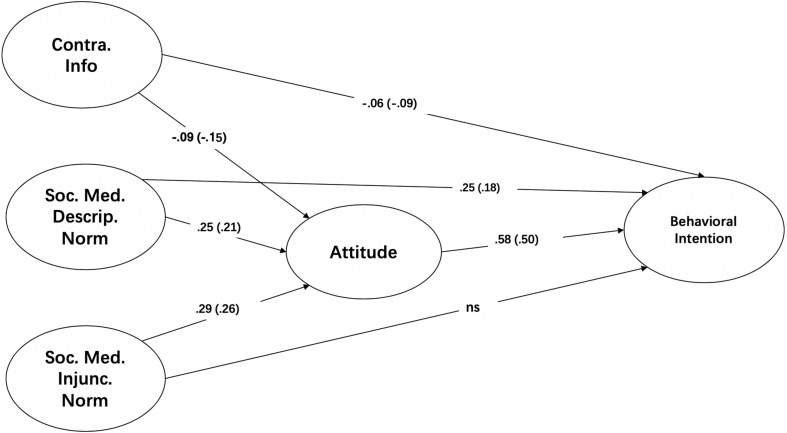
SEM for main effects.

A measurement model for the four latent variables was built for the pooled samples. Figure 2 shows that all fit indices of the measurement model met the cutoff points recommended by Hu and Bentler (1999) (χ^2^/*df* = 2.09, CFI = 0.99, RMSEA = 0.05, and SRMR = 0.03). Then, a structural model was built for the pooled samples, and the model fit statistics were found to be satisfactory (χ^2^/*df* = 1.70, CFI = 0.99, RMSEA = 0.04, and SRMR = 0.02).

The first four hypotheses analyze the relationships between predictors and behavioral intentions related to HPV vaccination. H1a predicts that greater exposure to contradictory HPV vaccine messages on social media is associated with a more negative attitude toward HPV vaccination. H1a was supported (B = −0.09, β = −0.15, *p* < 0.01). H1b predicts that greater exposure to contradictory vaccine messages on social media is associated with reduced intentions to receive the HPV vaccine. H1b was supported (B = −0.06, β = −0.09, *p* < 0.05).

H2a predicts that descriptive norms regarding HPV vaccination are positively associated with attitudes toward HPV vaccination. H2a was supported (B = 0.25, β = 0.21, *p* < 0.05). H2b predicts that descriptive norms on social media are positively related to behavioral intentions to receive the HPV vaccine. H2b was supported (B = 0.25, β = 0.18, *p* < 0.05). H3a predicts that injunctive norms regarding HPV vaccination are positively associated with attitudes toward HPV vaccination and examines the mediating role of attitudes. H3a was supported (B = 0.29, β = 0.26, *p* < 0.05). H3b predicts that injunctive norms on social media are positively related to behavioral intentions to receive the HPV vaccine. H3b was not supported (B = 0.10, β = 0.08, *p* = 0.39).

H4 predicts that attitudes toward HPV vaccination are positively associated with the intention to receive the HPV vaccine. H4 was supported (B = 0.58, β = 0.50, *p* < 0.001).

### Hypothesis Testing for Cultural Differences

The most important research question examined in this study concerns how China and the U.S. differ in the association between HPV vaccination-related contradictory information/norms on social media and intentions to receive the HPV vaccination by comparing corresponding path coefficients. The researchers analyzed measurement invariance before comparing regression coefficients and conducted three tests for configural, metric, and scalar invariance (Baumgartner and Steenkamp, 1998; Rensvold and Cheung, 2002). Configural invariance evaluates whether factor structures across groups are identical. According to the model fit indices shown in Table 4, configural invariance was established. The researchers then evaluated metric invariance, which measures whether constructs have the same meaning across groups. The researchers constrained the factor loadings of the measurement models for the two countries to make them identical. As Table 4 shows, compared to the fit indices of the baseline configural model, those of the metric invariance model did not deteriorate significantly, suggesting that the factor loadings were invariant between the two countries. Finally, to analyze scalar invariance, the researchers constrained both factor loadings and intercepts to make them identical. However, the model fits deteriorated under these constraints; thus, the intercepts were not invariant, and scalar invariance was not established. A comparison of regression paths does not require scalar invariance; it only requires metric invariance, which ensures equality across scale intervals (Baumgartner and Steenkamp, 1998, 2004).

**TABLE 4:** Fit indices for measurement invariance.

	*df*	χ^2^	Δχ^2^	CFI	ΔCFI	RMSEA	ΔRMSEA	Accept/reject
Configural invariance	140	246.64	NA	0.976	NA	0.063	NA	Accept
Full metric invariance	149	255.61	8.966	0.976	0	0.061	0.002	Accept
Full metric and scalar invariance	158	302.26	46.658	0.967	0.009	0.069	0.008	Reject

**FIGURE 3:**
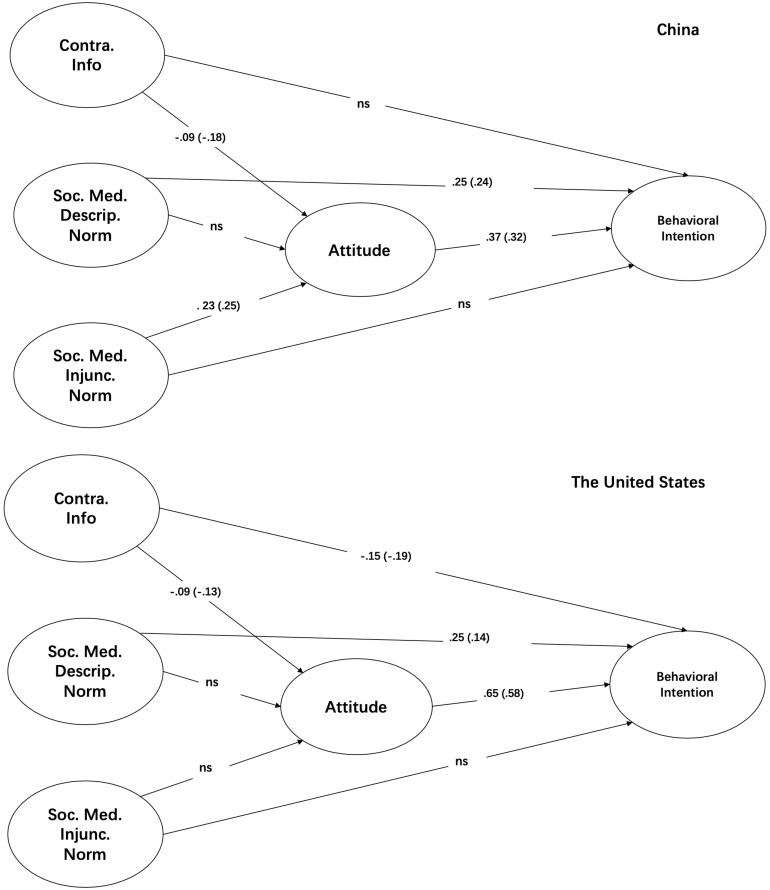
Multi-group SEM.

To answer the research question, the researchers operationally examined how the seven sets of path coefficients (the four hypotheses) varied between the samples from the two countries. In practice, the researchers built one model with all seven pairs of path coefficients constrained to make them equal and another model with one pair of path coefficients that were different from each other while the other six pairs of regression coefficients were made equal. Then, the researchers conducted a χ^2^ test with *df* = 1 to determine whether the two models differed significantly from each other, with a positive result denoting that the path coefficients differed across the two groups. In summary, of the seven pairs of path coefficients considered, three pairs were identical, and four were different (see Figure 3). The fit indices of the model that allowed three pairs of path coefficients to vary (χ^2^/*df* = 1.53, CFI = 0.98, RMSEA = 0.05, SRMR = 0.04) met the recommended cutoff points and were superior to those for the model in which all pairs of coefficients were constrained as equal (χ^2^/*df* = 1.62, CFI = 0.97, RMSEA = 0.06, and SRMR = 0.08) (Δχ^2^[4] = 25.23, *p* < 0.001). The specific tests and results are reported below. Additionally, the study controlled for whether the respondents lived with their partners/spouses (dummy), whether they were sexually active (dummy), and for their education level (not displayed in the figures). Only education level was found to be associated with attitudes and intentions regarding HPV vaccination.

The association between contradictory information exposure and attitudes (H1a) was the same across the two cultural groups (Δχ^2^_[__1__]_ = 2.19, *p* = 0.14); the non-standardized coefficient was -0.093 (*p* < 0.05). The relationship between descriptive norms on social media and attitudes (H2a) indeed varied between the two groups (Δχ^2^_[__1__]_ = 8.79, *p* < 0.01) but was not statistically significant for either group (China: B = 0.15, β = 0.16, *p* = 0.21; U.S.: B = 0.42, β = 0.25, *p* = 0.14). The association between injunctive norms on social media and attitudes (H3a) differed between the Chinese and U.S. samples (Δχ^2^_[__1__]_ = 8.15, *p* < 0.01): a positive relationship was found for the Chinese sample (B = 0.23, β = 0.25, *p* < 0.05) but not for the U.S. sample (B = 0.38, β = 0.25, *p* = 0.14).

The association between exposure to contradictory information and behavioral intention (H1b) differed between the Chinese and U.S. samples (Δχ^2^_[__1__]_ = 8.08, *p* < 0.01). Exposure to contradictory information was not related to behavioral intentions in the Chinese sample (B = −0.03, β = −0.05, *p* = 0.46), while for the U.S. respondents, exposure to contradictory information was negatively related to behavioral intentions (B = −0.15, β = −0.19, *p* < 0.01). The relationship between descriptive norms on social media and intentions (H2b) was identical across the two groups (Δχ^2^_[__1__]_ = 0.03, *p* = 0.87); the non-standardized and standardized coefficients were 0.25 and 0.24 (0.14 for the U.S.) (*p* < 0.05), respectively. The relationships between injunctive norms on social media and behavioral intentions (H3b) were also the same across the groups (Δχ^2^_[__1__]_ = 0.23, *p* = 0.62) and were not related (B = 0.10, β_cn_ = 0.11, β_us_ = 0.07, and *p* = 0.31).

The association between attitudes and behavioral intentions (H4) exhibited between-group differences (Δχ^2^_[__1__]_ = 12.55, *p* < 0.001). The magnitude of the coefficient of the association between attitudes and behavioral intentions for the Chinese sample (B = 0.37, β = 0.32, *p* < 0.001) was much smaller than that for the U.S. sample (B = 0.65, β = 0.58, *p* < 0.001).

## Discussion

The primary purpose of this study was to examine differences in how HPV vaccination-related contradictory messages and norms on social media are associated with young women’s attitudes and behavioral intentions regarding HPV vaccination in China and the U.S. Based on the results of a multigroup SEM, this study reveals interesting similarities and significant differences. The study finds that exposure to contradictory messages shows a stronger association with the intention to receive the HPV vaccination among American participants, while social approval (injunctive norms on social media) plays a role only for Chinese participants. The study also shows some similarities between the two cultural groups. First, the respondents’ attitudes are similarly associated with perceived contradictions in HPV vaccination-related messages; second, descriptive norms are related to behavioral intentions but not to attitudes. Possible explanations for the observed similarities and differences are provided below.

According to the descriptive statistics, the Chinese respondents have more favorable attitudes toward and stronger intentions to receive the HPV vaccine, and they report higher levels of exposure to contradictory information, descriptive norms, and injunctive norms regarding HPV vaccination than their U.S. counterparts. This might be because HPV vaccination is relatively new in China but not in the U.S. In 2018, the HPV vaccination rate in the U.S. was 51% among young people between the ages of 13 and 17 years (Mundell, 2019, August 22). However, although calls for the introduction of an HPV vaccine to the Chinese market were prevalent on Chinese social media for a few years prior, the China Food and Drug Administration waited until 2016 to approve the vaccine – 10 years after it became available in the U.S. Furthermore, reports show that the supply of HPV vaccines can hardly meet the huge demand in the Chinese market, and people must wait a long time to get the vaccine (Financial Times, 2018, June 24). This special situation in China might cause Chinese participants to have stronger expectations of receiving HPV vaccination than their U.S. counterparts and to have more positive attitudes toward the vaccine and stronger intentions to receive it. Meanwhile, because the HPV vaccination was introduced to China more recently, public discussion about this issue might be highly mixed. This may explain why the Chinese participants reported higher levels of exposure to contradictory messages about HPV vaccination. Alternatively, as our measures are self-reported, cultural differences in response styles may have been likely, and the respondents may have differed in their interpretations of the questions, which may lead to consistently higher scores on the Chinese side.

### Contradictory Information and Norms on Social Media

Among the hypothesized main effects, contradictory information promulgated through social media is indeed negatively associated with attitudes and behavioral intentions, while attitudes are positively associated with behavioral intentions. Attitudes were found to mediate the relationship between contradictory information exposure on social media and the intention to receive the HPV vaccination. Previous studies on media effects mostly indicate that people’s attitudes and intentions are affected in the direction in which media content directs them. For instance, Bond and Compton (2015) found that heterosexual television viewers tended to become more accepting of gay people as a result of media exposure to same-sex relationships. Dunn et al. (2015) correspondingly found that those who are often exposed to negative opinions are more likely to subsequently post negative opinions. However, very little research examines the effect of contradictory information exposure through media, especially through social media. The current study thus contributes to the literature by examining how mixed and contradictory information provided on social media is related to health-related attitudes and behavioral intentions.

The study found that injunctive norms on social media are positively associated with attitudes, which in turn are positively associated with behavioral intentions. That is, injunctive norms on social media and behavioral intentions are associated via attitudes. These findings, which resulted from pooling the data for the U.S. and Chinese respondents together, are in line with our predictions and with theories and previous empirical studies (e.g., Prislin and Wood, 2005; Rimal and Real, 2005; Jolley and Douglas, 2014; Dunn et al., 2017). However, the direct link between injunctive norms and social media and behavioral intentions is not significant.

### Cultural Similarities and Differences

The multigroup SEM found that the association between exposure to contradictory information on social media and attitudes vary little between the Chinese and U.S. respondents. This indicates that the attitudes are similarly related to contradictory messages for both groups. Media messages often change people’s behavioral intentions by changing their attitudes (Lee, 2011; Yoo and Tian, 2011). However, the present study found that this common indirect path does not exist when both groups are exposed to contradictory messages through social media. Contradictory messages were found to be directly associated with behavioral intentions among the U.S. participants but not among the Chinese participants. The U.S. participants may have had less tolerance for uncertainty and inaccuracy, as shown in previous studies (Hofstede, 2001), making them more likely to undergo peripheral processing of contradictory messages and directly respond to contradictory messages without their changing attitudes. The direct link found for the U.S. sample may also be attributed to the fact that while HPV vaccine use is in its infancy in China, and thus the Chinese population is less familiar with it, HPV vaccinations have been in use for a decade in the U.S., and American participants are thus more familiar with debates surrounding the vaccine. When faced with contradictory information on social media, the Chinese respondents may have had to undergo systematic information processing and evaluate the vaccine before they could form a behavioral intention. Additionally, the total effect of contradictory information exposure on behavioral intentions is much lower for the Chinese sample (−0.033 = −0.09 × 0.37) than that for the U.S. sample (−0.21 = −0.09 × 0.65 − 0.15). This suggests that contradictory messages may have had a stronger impact on the U.S. participants than on the Chinese participants. This suggests that U.S. health communication practitioners may consider paying more attention to contradictory messages when developing health promotion campaigns for social media and making authoritative information readily available to campaign targets.

Through the multigroup analysis, it was found that descriptive norms on social media play the same role in both groups. Upon seeing that other social media users have received the HPV vaccine, both the Chinese and American respondents were more inclined to do be vaccinated, and attitudes did not mediate this relationship in either group. In analyzing the main effects, it was found that descriptive norms on social media are not related to behavioral intentions overall but are related when the data are separated by group. This might be related to the non-equivalence of factor error variance between the two country groups. When we separated the data by country, this link became statistically significant. This finding indicates an interesting mechanism by which descriptive norms can be directly related to behavioral intentions without necessarily changing attitudes. Future research could further examine whether such mechanisms affect other human behaviors. The results also suggest that leveraging social influence through social media during an HPV communication campaign can supplement traditional mass communication-based campaign strategies (Noar and Head, 2011) that focus on the top-down delivery of health knowledge and information. Specifically, given the prevalence of social media use among the younger generation, health communication practitioners in both countries may consider motivating those who have received an HPV vaccination to share their experiences on social media to influence those who have not.

Additionally, injunctive norms perceived through social media were positively associated with behavioral intentions (via attitudes) only for the Chinese sample. Again, this shows the effects of collectivism versus individualism. Collectivistic cultures place greater pressures on individuals to identify with in groups than individualistic cultures (Triandis et al., 1988). Previous studies on social media and electronic word-of-mouth have revealed that more cohesive and intimate social relationships are more likely to form among Chinese social networking site users than among their American counterparts (Chu and Choi, 2011). As such, in China, which emphasizes collectivism, people may be more sensitive to fellow social media users’ approval or disapproval of HPV vaccination to avoid feeling alienated from the social media groups or communities to which they belong. Such results also suggest that future health studies, whether comparative or not, may consider measuring the nature of social media contacts. These differences can also have practical implications, and Chinese practitioners may consider encouraging people who have received the HPV vaccine to not only share their experience but also express approval of the vaccine, while this might not be necessary in the U.S.

## Limitations and Conclusion

This study has some limitations. First, some measurements could be further refined in future studies. Due to the study’s exploratory nature, it did not measure individual-level cultural beliefs; future studies can measure these beliefs to validate cultural differences between our two sample groups and to discover the precise mechanisms involved. The results of the study could be made more robust if individual access to health care and levels of insurance coverage for HPV vaccines were measured, as the two countries differ in terms of health care systems and insurance policies. This study measured perceived norms and attitudes using short scales, and future studies could use longer scales to improve reliability. Another variable, “exposure to contradictory messages,” was measured with one item, which might have introduced reliability problems and influenced the explanatory power of this variable. In terms of behavioral intentions, we failed to set a time frame for vaccination intent, which made it a weaker measurement in terms of tracking real differences in behavioral intent.

The study’s sample could be further improved. The study included only young female participants, limiting the generalizability of the study. We focused on this population because HPV vaccines are currently primarily provided to this group in China due to limited supplies. However, it would be worth examining cultural differences using a more diverse sample. Our data also show that more U.S. respondents than Chinese respondents were screened out of this study because Americans are less likely to be exposed to HPV vaccine-related information through social media. There might be two main reasons for this difference. First, the HPV vaccine is still a new issue for most Chinese due to its more recent availability in China, and this novelty may generate more discussion of the issue on social media. Chinese social media users may thus also be more motivated than their American counterparts to seek information related to the HPV vaccine. In addition, high social media penetration rates among young people in China (Clemen, 2020) and China’s limited medical resources for the general public may have led to fewer cases being removed from the Chinese sample.

The present study also has some other general limitations. First, the research model employed was not tested with a random sample, which might have affected the robustness of the results; thus, we should be cautious when generalizing them to the population at large. In addition, as our data are cross-sectional, we cannot establish causal relationships among variables. Considering the abovementioned limitations, we should be careful when generalizing the findings of this study to the broader population, such as females above the age of 26 years, males, or people from other cultures.

Despite these limitations, the present study still makes unique theoretical and practical contributions. Theoretically, the study expands the literature regarding the effects of contradictory information exposure and social norms on health attitudes and behaviors through the lens of social media, which has not yet been substantially studied. Moreover, the study reveals cross-cultural differences in how these factors are associated with intentions to receive the HPV vaccination. Since the U.S. population has been previously reported to show less tolerance for uncertainty than the Chinese population (Hofstede, 2001), the results of this study may indicate that contradictory messages disseminated through social media have a more negative association with intentions to receive the HPV vaccination for the U.S. sample; since China is more collectivistic, norms conveyed through social media may be a more important factor for the Chinese sample. More importantly, differences in the possible effects of contradictory messages and social norms between the two cultural contexts suggest that future studies can further pinpoint the exact mechanisms through which cultural beliefs affect HPV vaccination-related attitudes and behaviors.

Practically, the prevalence of unverified and flawed health information on social media has posed a great challenge to public health practitioners. Understanding how these factors impact health attitudes and behaviors is crucial for the planning and execution of health communication campaigns. Health institutions and social media platforms may work closely to identify how contradictory information about HPV vaccines is currently presented on social media and then make efforts to address confusion caused by such information by providing clear and easily understandable HPV information. Meanwhile, HPV communication interventions may also consider mobilizing those who have received the vaccine to share their experiences and express their endorsement so that pro-vaccination norms can be established on social media.

More importantly, cultural differences such as those identified in this study require practitioners to adopt culturally sensitive approaches when designing health campaigns for social media. For instance, contradictory messages can be more closely monitored when promoting HPV vaccines in the U.S., while encouraging those who have received the HPV vaccine to spread the word seems to be more important in China.

## Data Availability Statement

The raw data supporting the conclusions of this article will be made available by the authors, without undue reservation.

## Ethics Statement

The studies involving human participants were reviewed and approved by IRB of School of Journalism and Communication, Renmin University of China. The patients/participants provided their written informed consent to participate in this study.

## Author Contributions

SP responsible for conceptualization, questionnaire design, data collection, and writing literature review. DZ responsible for data analysis and writing method, result and discussion sections. JZ responsible for conceptualization, questionnaire design, writing discussion section, and editing.

## Conflict of Interest

The authors declare that the research was conducted in the absence of any commercial or financial relationships that could be construed as a potential conflict of interest.
